# Preliminary study on the application of contrast-enhanced ultrasound in lymphovenous anastomosis

**DOI:** 10.1016/j.jvsv.2025.102344

**Published:** 2025-10-14

**Authors:** Jing Wang, Min Lu, Mingxing Hu, Liuyi Li, Kui Zhang, Xu Li

**Affiliations:** a Department of Ultrasound Medicine, Bishan Hospital of Chongqing Medical University, Bishan Hospital of Chongqing, Chongqing, China; b Department of Lymphatic Microsurgery, Bishan Hospital of Chongqing Medical University, Bishan Hospital of Chongqing, Chongqing, China; c Department of General Internal Medicine, Fucheng Hospital of Chongqing, Chongqing, China

**Keywords:** Contrast-enhanced ultrasound, Indocyanine green (ICG), Lymphedema, Lymphovenous anastomosis (LVA)

## Abstract

**Objective:**

To explore the value of lymphatic contrast-enhanced ultrasound (CEUS) in lymphovenous anastomosis (LVA), with a particular focus on its role in preoperative lymphatic localization.

**Methods:**

On the basis of whether preoperative ultrasound lymphatic contrast imaging was performed, the lymphatic diameter and depth from the skin were measured. Patients were divided into two groups: indocyanine green (ICG, 21 patients) and ICG combined with CEUS (21 patients). The differences between the two groups were compared in terms of total surgery time; number of anastomoses; anastomosis time per lymphatic vessel; and limb volume reduction at 1 week, 1 month, and 3 months postoperatively. A *P* value of < .05 was considered to indicate statistical significance.

**Results:**

In the ICG + CEUS group, the success rate of ultrasound contrast imaging was 95%. The average lymphatic diameter was 0.6 ± 0.2 mm, and the average depth from the skin was 5.3 ± 2.8 mm. Among these patients, 74% had lymphatic reflux, 55% had reflux into the dermis, and 36% had collateral vessel formation. Compared with the ICG group, the ICG + CEUS group had a significantly shorter total surgery time (320 ± 124 vs 398 ± 93 minutes; *P* = .005), shorter anastomosis time per lymphatic vessel (14 ± 2 vs 32 ± 15 minutes; *P* < .001), and more anastomoses (median 19 vs 12; *P* < .001). There were no significant differences between the two groups in terms of limb volume reduction at 1 week, 1 month, or 3 months postoperatively (*P* > .05).

**Conclusions:**

CEUS can be a powerful supplement for locating lymphatic vessels before LVA.

**Article Highlights**

• **Type of Research:** Single-center retrospective cohort study
• **Key Findings:** In 42 patients with lymphedema undergoing lymphovenous anastomosis, the indocyanine green (ICG) + contrast-enhanced ultrasound (CEUS) group showed shorter total operative time (320 ± 124 vs 398 ± 93 minutes; *P* = .005), shorter anastomosis time per vessel (14 ± 2 vs 32 ± 15 minutes; *P* < .001), and more anastomoses (median 19 vs 12; *P* < .001) compared with the ICG-only group.
• **Take Home Message:** Preoperative CEUS combined with ICG enables more precise lymphatic mapping and significantly improves surgical efficiency in lymphovenous anastomosis.

Lymphedema is a chronic disease caused by the obstruction or lymphatic reflux, resulting in the accumulation of fluid in the soft tissue of the limb, secondary fibrous tissue hyperplasia, fatty sclerosis, and fascia thickening, manifested as limb edema, pain, or dysfunction.^1^ It can be categorized into primary and secondary types, with the latter caused primarily by acquired factors, such as surgery and infections. Studies have shown that, among patients with breast cancer who underwent lymph node dissection and postoperative radiotherapy, approximately 51% developed lymphedema within 4 years.^2^ In recent years, the incidence of gynecological malignancies has increased annually, leading to an increasing number of patients with lymphatic flow obstruction. This has further compromised the quality of life of patients with cancer and has garnered increased clinical attention.

Lymphovenous anastomosis (LVA) is an important surgical technique for treating lymphedema. This procedure reconstructs lymphatic-venous anastomoses, connecting the lymphatic vessels to low-pressure microveins and allowing the lymphatic fluid to directly drain into the blood circulation, thereby alleviating the symptoms of lymphedema. Accurate preoperative localization of functional lymphatic vessels is crucial for reducing the total operative time and increasing the success rate of surgery.^3^, ^4^, ^5^, ^6^, ^7^, ^8^ Indocyanine green (ICG) fluorescence imaging, currently the gold standard for preoperative lymphatic vessel localization in LVA, allows real-time visualization of lymphatic pathways via fluorescence signals. However, the effective imaging depth of ICG lymphography is generally 5 to 10 mm,^9^ although some reports have noted observations of up to 2 cm in selected scenarios.^10^ These limitations make it difficult to identify deeper lymphatic collectors, particularly in patients with dermal backflow, where the accumulation of fluorescent dye in the dermis may obscure underlying lymphatic vessels and hinder accurate localization and depth assessment.

High-frequency ultrasound has gained attention in recent years because of its advantages of being economical, convenient, nonradiative, and capable of visualizing the venous structures surrounding the lymphatic vessels. However, the average depth of lymphatic vessels is approximately 5.2 mm, and in cases of lymphedema, conventional high-frequency probes (9-14 MHz) struggle to distinguish lymphatic vessels, subcutaneous fluid accumulation, and surrounding small superficial veins, which affects the visibility of the lymphatic vessels.^11^, ^12^, ^13^ Some studies have confirmed that contrast-enhanced ultrasound (CEUS) can visualize lymphatic vessel pathways in real time and can be applied for preoperative lymphatic vessel localization in the LVA.^14^^,^^15^ However, this technique is still in the early stages of research. This study aimed to further validate its accuracy and feasibility in the preoperative localization of lymphatic vessels for LVA surgery, with the goal of optimizing surgical procedures and improving postoperative outcomes.

## Methods

### Subjects and outcomes

A retrospective study was conducted on 42 patients with lymphedema who were treated at the Bishan Hospital of Chongqing Medical University from January 2023 to November 2024 and underwent LVA surgery. The inclusion criteria were as follows:^1^ patients with a confirmed diagnosis of lymphedema^2^; patients who underwent LVA surgery^3^; patients who underwent preoperative ICG testing. The patients were divided into two groups according to whether lymphatic ultrasound imaging was performed preoperatively: the ICG group and the ICG + CEUS group. The exclusion criteria were as follows:^1^ patients with severe liver or renal dysfunction, respiratory failure, heart failure, active autoimmune or immunological diseases, or any other conditions that would make them unable to tolerate surgery^2^; patients with severe contrast agent allergies who could not tolerate ultrasound contrast imaging^3^; and pregnant or breastfeeding women. Ultimately, 21 patients were included in the ICG group, and 21 were included in the ICG + CEUS group.

This study was approved by the medical ethics committee of our hospital, and registered with the National Medical Research Registry (No.: MR-50-24-053,104). All patients provided written informed consent.

## Outcomes

The primary outcome was the total surgical duration (minutes) required for LVA.

Secondary outcomes included^1^ the number of anastomoses per patient,^2^ the anastomosis time per lymphatic vessel (minutes),^3^ and the limb-volume reduction rate at 1 week, 1 month, and the 3-month follow-up.

### CEUS

A Philips EPIQ 5 color Doppler ultrasound diagnostic system equipped with a high-frequency linear array probe with a frequency range of 4 to 18 MHz was used. High-frequency B-mode and color Doppler ultrasound were used to identify superficial recipient veins, whereas CEUS was employed exclusively for lymphatic visualization and evaluation of lymphatic flow. The ultrasound contrast agent was SonoVue (Bracco, lyophilized powder: 25 mg, SF6 microbubbles: 59 mg), which was reconstituted with 5 mL of physiological saline to form a suspension and shaken well before use. The procedure was performed without any local or systemic anesthesia, as it involved only brief subcutaneous injections and was well-tolerated by all patients. Next, 0.5 mL of the contrast agent suspension was injected into the following areas via a 21G needle: for the lower limb examination, the first and fourth toe webs, the medial and lateral aspects of the ankle joint; for the upper limb examination, the first and fourth interdigital webs, the medial and lateral aspects of the wrist joint.

CEUS was performed before ICG lymphography because the microbubble enhancement lasts only a short time and is not suitable for prolonged intraoperative monitoring. Performing CEUS first also allows accurate preoperative localization and depth measurement of lymphatic vessels—particularly valuable in patients with dermal backflow where ICG fluorescence may not clearly show deeper lymphatics.

ICG, which provides longer-lasting fluorescence, was therefore used subsequently for continuous intraoperative guidance and assessment of anastomotic patency.

**Fig 1:**
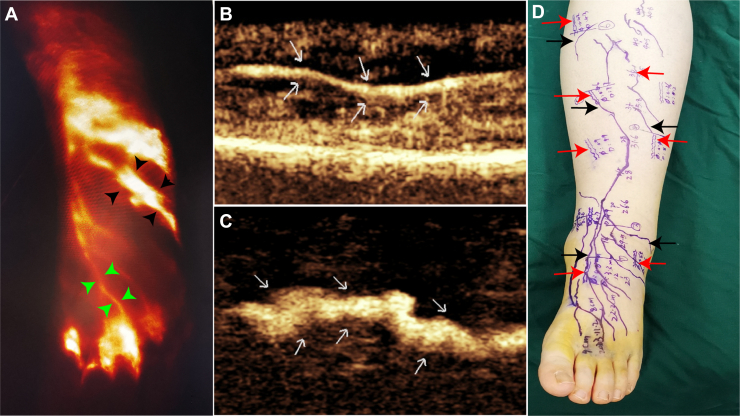
Localization of lower-limb lymphatic vessels in a stage II secondary-lymphedema patient using indocyanine green (ICG) and contrast-enhanced ultrasound (CEUS) before lymphovenous anastomosis (LVA) surgery. **(A)** ICG image of the dorsum of the foot showing lymphatic vessels (*green and black short arrows*). The *green arrow* indicates a lymphatic vessel with normal diameter and course, whereas the *black arrow* indicates a dilated lymphatic vessel. **(B)** CEUS image at the *green arrow* site in panel **(A)** showing a lymphatic vessel with normal course and no dilation (*white arrow*). **(C)** CEUS image at the *black arrow* site in panel **(A)** showing a tortuous, dilated lymphatic vessel. **(D)** Surface markings of the lymphatic vessels: *black arrows* indicate ICG-based markings, and *red arrows* indicate CEUS-based markings.

The patient was positioned in the supine position, with the probe held lightly and without applying pressure to avoid compressing the lymphatic vessels. The superficial organ ultrasound contrast mode was activated, with the depth adjusted to 2.5 cm. A low mechanical index (MI = 0.06) imaging technique was used, with the focal point set at the far field to minimize microbubble destruction. The dynamic range was maximized at 72 MHz to reduce the suppression of tissue signals. After injection, the injection site and drainage area were gently massaged with a cotton swab or fingers to promote contrast medium dispersion. The functional lymphatic vessels were then identified, and their morphology was assessed for signs of distortion, dilation, interruption, or formation of collateral branches. Any retrograde flow of contrast medium within the lymphatic vessels or from superficial lymphatic vessels to the skin was also observed and documented qualitatively as present or absent. The diameter and depth of each lymphatic vessel were measured on frozen CEUS images using the electronic calipers of the ultrasound system, and reference marks were drawn on the skin surface (Fig 1).

### ICG

The same injection sites used for ultrasound contrast imaging were used, with 0.5 mL (2.5 mg/mL) of ICG injected subcutaneously via a 21G needle. Thirty minutes after the injection, a PDE infrared imaging system was used to excite ICG fluorescence in the subcutaneous tissue. The functional lymphatic vessels were identified, and their locations were marked on the skin to define the surgical incision boundaries (Fig 1).

### LVA surgical operation

**Fig 2:**
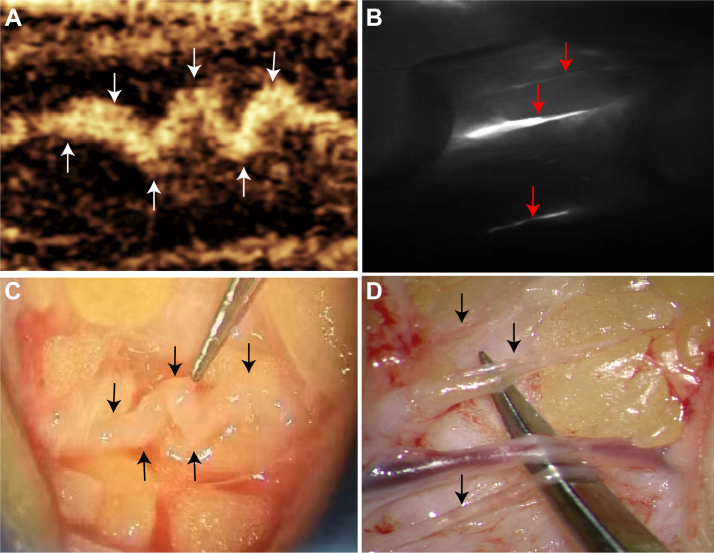
Contrast-enhanced ultrasound (CEUS) and indocyanine green (ICG) images of lymphatic vessels in a patient with stage II secondary lymphedema and corresponding intraoperative lymphovenous anastomosis (LVA) findings. **(A)** CEUS showing an “M-shaped” lymphatic vessel (*white arrow*). **(B)** ICG showing multiple parallel lymphatic vessels (*red arrow*). **(C)** Intraoperative micrograph corresponding to panel **(A)** demonstrating the “M-shaped” lymphatic vessel (*black arrow*). **(D)** Intraoperative micrograph corresponding to panel **(B)** demonstrating multiple parallel lymphatic vessels (*black arrow*).

**Fig 3:**
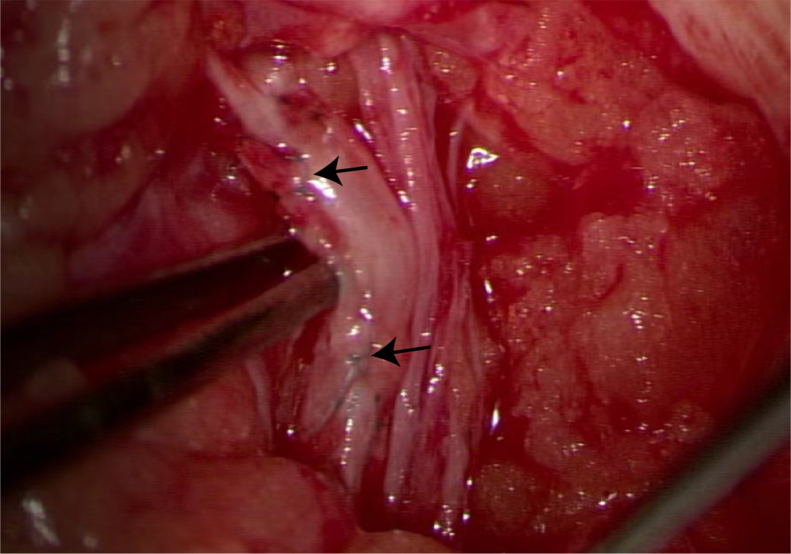
Bilateral lymphovenous anastomosis (LVA). Micrograph illustrating the bilaterally anastomosed surgical technique (*black arrow* indicates the anastomosis between the lymphatic vessel and the vein).

**Fig 4:**
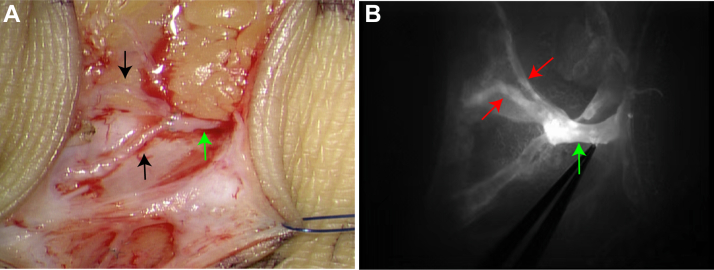
Indocyanine green (ICG) assessment of lymphovenous anastomosis (LVA) patency. **(A)** Intraoperative micrograph showing two lymphatic vessels anastomosed to an adjacent vein in a “Y-shape” (*black arrow* indicates the lymphatic vessel; *green arrow* indicates the vein). **(B)** ICG image demonstrating patency of the anastomosis between the lymphatic vessel and the adjacent vein (*red arrow* indicates the lymphatic vessel; *green arrow* indicates the vein).

In the ICG group, surgery was performed on the basis of the fluorescence imaging markings, whereas both the fluorescence and ultrasound contrast imaging sites were explored during surgery in the ICG + CEUS group (Fig 2). Intraoperatively, depending on the location, number, diameter, and other characteristics of the lymphatic vessels and surrounding superficial veins, end-to-end anastomosis or end-to-side anastomosis was performed. If retrograde flow was detected in the lymphatic vessels via ultrasound contrast imaging, bidirectional anastomosis was performed, where both segments of the incised lymphatic vessel were anastomosed end-to-side with the superficial veins (Fig 3). After the anastomosis was completed, the surgical area was exposed to fluorescence lighting. Successful anastomosis was confirmed when ICG-stained lymph fluid drained smoothly from the lymphatic vessels into the veins (Fig 4). The surgery was performed by a single surgeon with over 10 years of microsurgical experience.

All anastomoses were performed under an operating microscope using interrupted 11-0 nylon sutures.

### Limb-volume measurement and reduction rate

Circumferential measurements of the affected limb were obtained at 10-cm intervals, starting from the tip of the toes (or fingers) to the groin (or axilla). The limb was divided into consecutive truncated cones, and the total limb volume (V) was calculated using the truncated-cone formula:^16^

$$V=\sum \frac{\pi \times h}{12}\times \left(C_{1}^{2}+C_{1}\times C_{2}+C_{2}^{2}\right)$$

where h is the segment length (10 cm) and C_1_ and C_2_ are the circumferences at the distal and proximal ends of each segment.

The volume-reduction rate (VRR) at each follow-up was calculated as

$$\text{VRR}\left(\%\right)=\frac{V_{\text{pre}}-V_{\text{post}}}{V_{\text{pre}}}\times 100$$

where V_pre_ is the preoperative total limb volume and V_post_ is the postoperative.

Measurements were obtained once at each time point—preoperatively and at 1 week, 1 month, and at a 3-month follow-up—by two experienced physicians working together.

## Statistical analysis

Data analysis was conducted via SPSS version 27.0 (IBM Corp). The Shapiro-Wilk test was applied to assess the normality of all continuous variables.

Normally distributed variables are presented as mean ± standard deviation (SD) and compared between groups using the independent samples *t*-test; within-group changes were analyzed with the paired-samples *t*-test. Non-normally distributed variables, including total operative time, anastomosis time per vessel, and the 1-month limb-volume reduction rate (which did not meet the normality assumption in one group), are expressed as median (interquartile range) and compared between groups using the Mann-Whitney *U* test. Categorical variables are presented as counts (percentages) and compared using the χ^2^ test or Fisher’s exact test when appropriate. A two-tailed *P* value < .05 was considered statistically significant.

## Results

### General conditions

In total, 42 patients with lymphedema who met the inclusion criteria were enrolled, with 21 patients in the ICG group and 21 in the ICG + CEUS group. Among these patients, there were five cases of primary lymphedema and 37 cases of secondary lymphedema (4 cases of breast cancer, 10 cases of endometrial cancer, 15 cases of cervical cancer, 5 cases of ovarian cancer, 1 case of liver cancer, 1 case of malignant melanoma in the groin region, and 1 case of thoracic duct stenosis). The average age of the patients was 56 ± 11 years (range, 34-79 years), and 37 females and 5 males were included. Notably, 12 patients had diabetes, and 22 had hypertension. Clinical staging of lymphedema was determined according to the International Society of Lymphology (ISL) staging system^17^; there was one patient in stage I, 35 in stage II, and five in stage III. The initial locations of lymphedema included two cases in the perineum, 35 cases in the lower limbs, and five cases in the upper limbs. The detailed preoperative clinical characteristics of the patients are presented in Table I. There were no statistically significant differences in age, body mass index (BMI), hypertension, diabetes, duration of lymphedema, or ISL clinical stage between the ICG group and the ICG + CEUS group, with all *P* values greater than .05.

**Table I:** Baseline clinical characteristics of patients in the ICG and ICG + CEUS groups

Variable	ICG group (n = 21)	ICG + CEUS group (n = 21)	*P*
Age, years	56 ± 10	57 ± 12	.770^a^
BMI, kg/m^2^	27.3 (24.2-27.7)	26.8 (26.1-30.4)	.28^b^
Hypertension	11 (52)	11 (52)	1.000^b^
Diabetes mellitus	7 (33)	5 (24)	.500^b^
ISL clinical stage	2 (2-2)	2 (2-2)	.268^b^
Duration of lymphedema, years	3 (1-7)	5 (2-7)	.393^b^

*BMI,* Body mass index; *CEUS,* contrast-enhanced ultrasound; *ICG,* indocyanine green; *ISL,* International Society of Lymphology.

Data are presented as number (%), mean ± standard deviation, or median (interquartile range).

a Independent samples *t* test.

b Mann-Whitney *U* test.

### Findings from lymphatic CEUS

**Fig 5:**
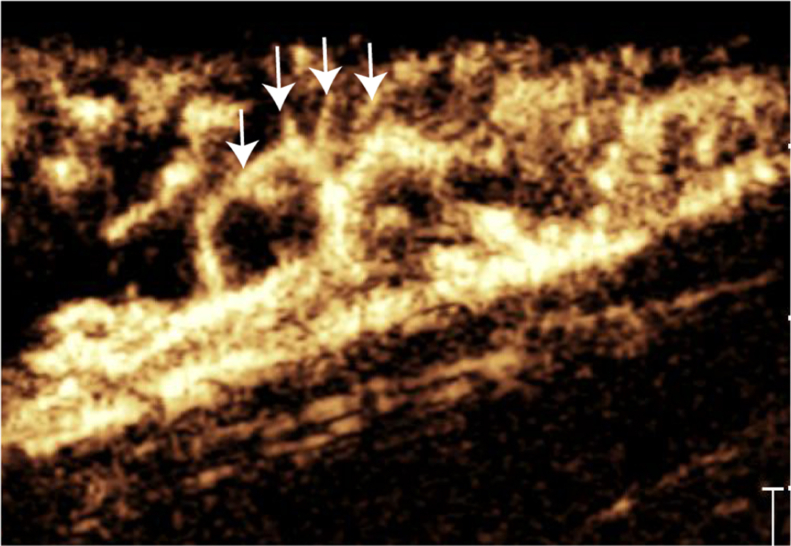
The contrast-enhanced ultrasound (CEUS) image of a lymphatic communicating branch, with the *white arrow* indicating the communicating branch.

In the ICG + CEUS group, CEUS successfully visualized lymphatic vessels in all patients except one with primary lymphedema, yielding a CEUS visualization success rate of 95%; intraoperative ICG visualization was not quantified in this study. Overall, 119 lymphatic vessels were localized preoperatively, with an average diameter of 0.6 ± 0.2 mm (range, 0.3-3.2 mm) and an average depth from the skin of 5.3 ± 2.8 mm (range, 0.9-16.6 mm). Among these vessels, 88 (74%) exhibited reflux within the lymphatic vessels, 65 (55%) showed reflux into the dermal layer (Supplementary Video, online only), and 43 (36%) collateral (communicating) lymphatic channels (Fig 5).

### Comparison of surgery-related indicators between the ICG group and the ICG + CEUS group

The mean duration of follow-up was 3.2 ± 0.8 months (range, 2-4 months). Compared with the ICG Group, the ICG + CEUS group demonstrated significantly shorter total operative time, more anastomoses, and shorter anastomosis time per lymphatic vessel (*P* < .01 for all). However, no significant differences were found in limb-volume reduction rates at 1 week, 1 month, or 3 months postoperatively (*P* > .05 for each comparison), as detailed in Table II.

**Table II:** Comparison of operative and postoperative outcomes between the ICG and ICG + CEUS groups

Variable	ICG group (n = 21)	ICG + CEUS group (n = 21)	*P*
Total operation time, minutes	370 (340-462)	280 (245-346)	.005^a^
No. anastomosis	12 (10-17)	19 (18-31)	<.001^a^
Anastomosis time per vessel, minutes	32 ± 15	14 ± 2	<.001^b^
Limb-volume reduction at 1 week, %	7.8 ± 5.2	7.2 ± 3.5	.671^b^
Limb-volume reduction at 1 month, %	7.7 (5.2-10.8)	8.3 (6.2-10.1)	.960^a^
Limb-volume reduction at 3 months, %	10.2 ± 4.9	10.1 ± 4.1	.894^b^

*CEUS,* Contrast-enhanced ultrasound; *ICG,* indocyanine green.

Data are presented as mean ± standard deviation or median (interquartile range).

a Mann-Whitney *U* test.

b Independent samples *t* test.

## Discussion

LVA is a safe and effective treatment for lymphedema, significantly reducing the severity of the condition and lowering the incidence of cellulitis, especially in the early stages of the disease.^18^, ^19^, ^20^ During LVA, ICG fluorescence imaging is widely used as a standard method for visualizing the lymphatic system. ICG, a tricarbocyanine dye, serves as a contrast agent that, upon entering the body through lymphatic vessels, binds to proteins in lymph fluid to form complexes that emit fluorescence when stimulated by near-infrared light. This fluorescence signal provides real-time imaging of the lymphatic pathways, assisting surgeons in locating lymphatic vessels during the procedure while enabling immediate postoperative assessment of anastomosis drainage effectiveness. However, in patients with subcutaneous reflux, the fluorescence from ICG may diffuse within superficial tissues, severely hindering the visualization of lymphatic vessels and making it difficult to accurately trace the paths of deeper functional vessels.^15^

Our study revealed that approximately 55% of lymphatic vessels exhibited subcutaneous (dermal) reflux, and 36% presented with collateral (communicating) lymphatic channels, making localization with ICG alone challenging. CEUS, which is not affected by dermal reflux, clearly delineates superficial lymphatic vessels across all layers and provides precise depth measurements. We observed an average lymphatic-vessel depth of 5.3 ± 2.8 mm (range, 0.9-16.6 mm), consistent with Hara et al,^4^ and beyond the ∼1.5 cm detection limit of ICG lymphography.^9^^,^^10^ Preoperative CEUS significantly improved operative efficiency, as reflected by shorter operative time, reduced anastomosis time per vessel, and a greater number of anastomoses compared with ICG alone, in agreement with the findings of Jia Zhu et al.^21^ CEUS is particularly valuable for accurately localizing lymphatic vessels, which in turn enhances surgical efficiency, and is especially beneficial in patients with dermal reflux; therefore, CEUS is recommended for all patients prior to LVA surgery.

During the CEUS process, 74% of the lymphatic vessels demonstrated retrograde flow of sulphur hexafluoride microbubbles, suggesting lymphatic valve incompetence. Because reflux may adversely affect postoperative drainage, as supported by Yoshida et al,^22^ when reflux was present, we used bidirectional anastomosis to enhance lymphatic fluid drainage. Despite these operative advantages, CEUS did not yield a significant difference in early postoperative limb-volume reduction compared with ICG alone. Potential explanations for this may include variations in the severity of lymphedema among patients, differences in postoperative rehabilitation, therapy adherence, daily activity level, and anastomosis techniques. Additionally, the relatively small sample size and short follow-up period in this study, combined with a limited range of postoperative efficacy indicators, may have masked early clinical benefits.

CEUS has certain technical limitations, such as a narrow detection range and a short imaging duration,^23^ necessitating multiple injections of the contrast agent. Additional limitations of this study include the sequential enrollment of the ICG group before the CEUS group—introducing potential learning-curve bias—as well as the single-center design, small sample size, relatively short follow-up, and a limited range of postoperative efficacy indicators.

## Conclusions

CEUS appears to be a promising adjunct for preoperative lymphatic vessel localization in LVA, offering the potential to improve surgical efficiency through assessment of lymphatic vessel function and accurate measurement of vessel diameter and depth; however, larger multicenter studies with longer follow-up are needed to confirm these preliminary findings before broad clinical adoption. Additionally, we recommend performing LVA in the early stages of lymphedema to promptly reconstruct lymphatic return pathways, thereby preventing excessive dilation and reflux of lymphatic vessels and reducing patients’ reliance on long-term compression therapy.

## Author Contributions

Conception and design: XL, JW, ML

Analysis and interpretation: XL, JW, ML, ZK

Data collection: XL, JW, XH, LL

Writing the article: XL, JW, XH

Critical revision of the article: XL, JW, XH, LL, ML, ZK

Final approval of the article: XL, JW, XH, LL, ML, ZK

Statistical analysis: XL, ZK

Obtained funding: JW

Overall responsibility: XL

JW and ML contributed equally to this article and share co-first authorship.

## Funding

This work was supported by the Social and Livelihood of Science and Technology project of Chongqing Bishan District Science and Technology Bureau, Chongqing of China (Grant No. BSKJ2024045). The Social and Livelihood of Science and Technology project of Chongqing Bishan District Science and Technology Bureau had no involvement in the study design or collection, analysis, and interpretation of data. The Social and Livelihood of Science and Technology project of Chongqing Bishan District Science and Technology Bureau was not involved in the decision to submit the manuscript for publication. The Youth Scientific Research and Innovation Team of Bishan Hospital of Chongqing, Bishan Hospital of Chongqing Medical University (Grant No. BYKY-CX2024012).

## Disclosures

None.

## Appendix (online only)

Supplementary Video (online only) Contrast-enhanced ultrasound (CEUS) of the right upper limb in a patient with stage II lymphedema, demonstrating intralymphatic reflux and dermal backflow. The lymphatic vessel is indicated by the *white arrow*. Before 8 seconds, sulfur hexafluoride microbubbles flow from right to left within the lymphatic vessel; after 8 seconds, the direction reverses, with microbubbles flowing from left to right into the dermis.
